# Preschoolers' Helping Motivations: Altruistic, Egoistic or Diverse?

**DOI:** 10.3389/fpsyg.2021.614868

**Published:** 2021-04-13

**Authors:** Jian Hao, Xu Du

**Affiliations:** Beijing Key Laboratory of Learning and Cognition, School of Psychology, Capital Normal University, Beijing, China

**Keywords:** prosocial behavior, altruistic motivation, egoistic motivation, helping, preschooler

## Abstract

Based on Eisenberg et al.'s model of prosocial motivations, the present study examined what motivates preschoolers to display instrumental helping and how various motivations develop during the preschool years. The participants were 477 preschoolers aged 3–5 years assigned to one of five groups. In each experimental group, the experimenter emphasized an altruistic or egoistic helping motivation, namely, empathic concern, moral rules, praise or rewards. In the control group, no helping motivations were emphasized. Their instrumental helping was then measured by sorting cards for a sick child to play a game. The results show that each helping motivation had a positive effect on instrumental helping. Most of the motivational effects were similar across age, but the motivational effect of empathic concern increased obviously at the age of 5 years. Therefore, the present study reveals that both altruistic and egoistic motivations motivate preschoolers to help others. Most of the motivations develop steadily during the preschool years, but empathic concern as an altruistic motivation increases greatly at the end of the preschool years. The present study thus confirms the diversity of preschoolers' helping motivations with Eisenberg et al.'s model of prosocial motivations.

## Introduction

Prosocial behavior increases with age during the preschool years (Hart et al., 1992; Persson, 2005; Gummerum et al., 2010; Van Berkel et al., 2015). Meta-analytic and cross-cultural studies have found that prosocial behavior is associated with many important factors, including theory of mind (Imuta et al., 2016; Cowell et al., 2017), executive functions (Cowell et al., 2017), fairness preference (Huppert et al., 2019), and maternal education (Cowell et al., 2017). These studies suggest that preschoolers have increasing abilities to display prosocial behavior. However, prosocial behavior is defined as voluntary behavior intended to benefit others (Eisenberg, 2010; Eisenberg et al., 2010). This definition emphasizes motivations for prosocial behavior, namely, concern for others. Thus, it is also important to explain the development of preschoolers' prosocial behavior from motivations. Whether preschoolers' prosocial behavior is increasingly motivated by concern for others' welfare should be clarified.

Prosocial behavior takes multiple forms, including comforting, sharing, informing, and instrumental helping (Warneken and Tomasello, 2009). Infants have displayed different forms of prosocial behavior (Svetlova et al., 2010; Dunfield et al., 2011). However, from the second year of life, they display instrumental helping more frequently than they show other types of prosocial behavior (Svetlova et al., 2010; Dunfield and Kuhlmeier, 2013). Instrumental helping involves acting on behalf of others who are unable to achieve their goals (Warneken and Tomasello, 2009). Recent reviews discuss the motivations for young children's prosocial behavior, especially instrumental helping (Hepach et al., 2013; Warneken, 2015; Hepach, 2017). Researchers argue that young children do not seem to express empathy or sympathy when providing instrumental help (Hepach, 2017). Accordingly, researchers are curious about what motivates young children's instrumental helping.

Researchers have proposed that prosocial behavior may rely on various motivations. Based on developmental psychology, social psychology, and economics, the concepts of psychological egoism and homo economicus are considered to reflect egocentric motivations for prosocial behavior (Haski-Leventhal, 2009). Because people care about humanity, prosocial behavior is also considered other-centric (Haski-Leventhal, 2009). According to evolutionary theories, prosocial behavior involves various proximate and ultimate mechanisms, such as reciprocity, concern for praise and blame, kin selection, and group selection (Nowak, 2006; Scott-Phillips et al., 2011).

Recently, based on Batson's (2011) work, Eisenberg et al. (2016) proposed a heuristic model of prosocial motivations. They summarized various motivations for prosocial behavior on a continuum from altruistic to egoistic motivation. Empathic concern and adherence to internalized principles are two typical altruistic motivations that emphasize “motivated by the ultimate goal of increasing another's welfare” (Eisenberg et al., 2016, p. 1669). Obtaining rewards and obtaining approval are two typical egoistic motivations that emphasize “motivated by the ultimate goal of increasing one's own welfare” (Eisenberg et al., 2016, p. 1669). This model suggests that motivations for prosocial behavior can be better investigated on a continuum from altruistic to egoistic. By reviewing previous studies, Eisenberg et al. (2016) indicated the existence of both altruistic and egoistic motivations in children's prosocial behavior. Batson (2011) also claimed that altruistic and egoistic motivations can simultaneously exist. We agree with these perspectives because caring about others' welfare does not mean completely ignoring one's own welfare. Children may be better equipped to help others if their own welfare is assured.

Although Eisenberg et al.'s (2016) model provides a theoretical framework for investigating prosocial motivations, some issues are still not clear and need to be further clarified. First, according to Eisenberg et al.'s (2016) model, what motivates 3–5-year-old preschoolers to display instrumental helping, altruistic, egoistic or diverse motivations? Second, how do various helping motivations develop during the preschool years? Previous reviews argue that young children's instrumental helping is motivated by a concern for others' welfare based on physiological evidence and evolutionary theories (Hepach et al., 2013; Warneken, 2015; Hepach, 2017). Meanwhile, classic developmental perspectives that young children are egocentric (Piaget, 1950) should also be noted. These various perspectives seem to suggest that both altruistic and egoistic motivations may exist in the preschool years. Consistent with this inference, previous studies have found that hedonism and the needs of others are most frequently mentioned in preschoolers' moral reasoning (Eisenberg et al., 1983, 1987). Hedonistic reasoning reflects a concern with self-oriented consequences such as personal gains, whereas needs-oriented reasoning reflects a concern for others' physical, material, and psychological needs. Therefore, it is reasonable to use Eisenberg et al.'s (2016) model to further verify the diversity of preschoolers' helping motivations.

Empathic concern is an important altruistic motivation in Eisenberg et al.'s (2016) model. Empathy enables individuals to understand the feelings of others and respond in a sensitive manner (Taylor et al., 2013). By reviewing previous studies, Eisenberg et al. (2016) presented evidence that even toddlers can show prosocial behavior due to empathy. Although empathy is widely acknowledged to be related to altruism (Eisenberg et al., 2006), it is not considered to drive toddlers' instrumental helping (Warneken, 2015). A recent study found that an experimenter's sad or neutral emotional expression did not affect toddlers' instrumental helping (Newton et al., 2014). Newton et al. (2014) explained that although very young children could empathize with others, empathy might not lead them to focus on others' needs. When entering the preschool years, things may be different. Theory of mind as an ability to understand mental states significantly develops during the preschool years (Wellman et al., 2001). When empathizing with others, preschoolers may be able to further think about others' needs or desires so that they can provide useful help. Moreover, a number of studies have demonstrated that empathy is an important driver of prosocial roles, dispositional prosocial behavior or prosocial behavior in the preschool years (Lennon et al., 1986; Holmgren et al., 1998; Howe et al., 2008; Belacchi and Farina, 2012). Thus, empathic concern is likely to motivate preschoolers' instrumental helping.

Adherence to internalized principles is another important altruistic motivation in Eisenberg et al.'s (2016) model. It has been found that prosocial behavior in adults is primarily driven by moral preferences for doing the right thing (Capraro and Rand, 2018; Tappin and Capraro, 2018; Capraro and Vanzo, 2019; Capraro et al., 2019). Eisenberg et al. (2016) argued that children are less likely to be motivated by abstract moral principles but may be affected by moral rules such as equality and fairness in prosocial decision making. According to Kohlberg (1963), there are differences between moral principles and moral rules: moral principles, such as the principle of conscience, are social ideals that are abstracted from various concrete moral rules; by contrast, moral rules are concrete rules for actions. What preschoolers are able to acquire is more likely moral rules rather than moral principles. Previous studies have shown that preschoolers' awareness of the rule of fairness is manifested in their discussion of hypothetical scenarios (Hod-Shemer et al., 2018) and allocation of rewards and punishments (Rakoczy et al., 2016; Smith and Warneken, 2016). Furthermore, preschoolers' moral judgements or reasoning are closely associated with their prosocial behavior (Eisenberg-Berg and Hand, 1979; Miller et al., 1996; Ongley et al., 2014). Because one's moral judgements or reasoning essentially reflect moral rules he or she adheres to (Kohlberg, 1969), previous studies suggest that moral rules may motivate preschoolers' prosocial behavior. A moral rule that “it is right to help others” may motivate preschoolers' instrumental helping.

Obtaining approval, which generally involves being praised, is an important egoistic motivation in Eisenberg et al.'s (2016) model. Praise can enhance feelings of autonomy, competence, and self-efficacy (Henderlong and Lepper, 2002), consequently benefiting the self. Social approval from peers is less valued by children than by adolescents (Eisenberg et al., 2016). Previous studies have mainly investigated the effect of praise from adults on young children's prosocial behavior (Guttmann et al., 1985; Warneken and Tomasello, 2008; Ulber et al., 2016). By reviewing previous studies, Warneken (2015) concluded that children younger than 5 years may not care about the presence of others when behaving prosocially, implying that praise may not motivate those children's prosocial behavior. Consistent with this conclusion, Warneken and Tomasello (2008) reported that 20-month-olds displayed equivalent levels of instrumental helping after experiencing praise and neutral feedback. Ulber et al. (2016) found that praise did not increase 3-year-olds' subsequent sharing behavior. In contrast, Guttmann et al. (1985) found that a higher percentage of 4–5-year-olds provided instrumental help after they were told that they would be praised for helping. Therefore, praise from adults may motivate instrumental helping in 5-year-olds rather than younger preschoolers.

Obtaining rewards is another important egoistic motivation in Eisenberg et al.'s (2016) model. According to behaviorism, rewards can reinforce behavior. When confronted with rewards, children display prosocial behavior to obtain them. Guttmann et al. (1985) found that a higher percentage of 4–5-year-olds provided instrumental help after they were told that they would receive tangible rewards for helping, supporting the perspective of behaviorism. Nevertheless, some studies yield different findings. Warneken and Tomasello (2008) reported that 20-month-olds showed less instrumental help after they were rewarded for their previous instrumental helping. Likewise, Ulber et al. (2016) indicated that rewards for previous sharing behavior undermined 3-year-olds' subsequent sharing behavior. There are essential differences between Guttmann et al.'s (1985) study and the latter studies. In Guttmann et al. (1985), preschoolers were told that they would obtain rewards if they helped. In this circumstance, more preschoolers displayed instrumental helping. In the latter studies, young children were first required to show prosocial behavior and were then immediately rewarded. This procedure induced them to attribute their prosocial behavior to a desire for rewards. Only under this circumstance were their subsequent prosocial behavior undermined. As the “overjustification” hypothesis claims, “a person's intrinsic interest in an activity may be undermined by inducing him to engage in that activity as an explicit means to some extrinsic goal” (Lepper et al., 1973, p. 130). Generally, rewards can motivate prosocial behavior; however, if children are induced to attribute their prosocial behavior to a desire for rewards, rewards show negative effects. In the present study, the question to be answered was whether rewards could motivate preschoolers' instrumental helping. Because it did not involve any attributional processes, a positive effect of rewards is expected.

In summary, based on previous studies, both altruistic and egoistic motivations may motivate preschoolers' instrumental helping. In addition, developmental trends of altruistic and egoistic motivations in the preschool years should also be clarified. Previous studies have not clearly portrayed how various helping motivations develop during the preschool years. Some studies indicate that from the end of the preschool years to elementary school years, children's other-oriented reasoning increases, and their hedonistic reasoning decreases (Eisenberg et al., 1983, 1987). Based on the developmental trends of altruistic and egoistic orientations in moral reasoning, it is reasonable to expect that the effects of altruistic and egoistic motivations on instrumental helping may also reveal similar developmental trends. However, obtaining praise may be an exception. As mentioned above, the effect of praise on instrumental helping may exist only in 5-year-olds. In other words, this egoistic motivation may not develop until the age of 5 years.

The present study aimed to investigate the motivations for preschoolers' instrumental helping under the theoretical framework of Eisenberg et al. (2016). Two research questions were proposed. First, what motivates preschoolers to display instrumental helping? Second, how do various helping motivations develop during the preschool years? Three- to five-year-olds were assigned to one of four experimental groups or a control group. In each experimental group, the experimenter emphasized a specific helping motivation, namely, empathic concern, moral rules, praise, or rewards. In the control group, the experimenter did not emphasize any helping motivations. The preschoolers were then given an opportunity to provide instrumental help. We hypothesized that empathic concern, moral rules and rewards would motivate all age groups of preschoolers to display instrumental helping, but praise would motivate only 5-year-olds' instrumental helping. In addition, altruistic helping motivations increase and egoistic helping motivations decrease during the preschool years. Obtaining praise may not develop as an egoistic motivation until the age of 5 years.

## Materials and Methods

### Participants

The participants were recruited from seven preschools in cities of Hebei Province in China. The 477 participants were 3–5-year-old preschoolers. They were randomly assigned to one of five groups. The sample size, gender and age information of each group are shown in Table 1. Informed consent was obtained from the parents of the preschoolers, and the study was approved by the Research Ethics Board of the School of Psychology of Capital Normal University.

**Table 1:** The sample size, gender, and age (months) of each group.

		***N***	**Female/Male**	***M*_**age**_ (*SD*)**	**Range**
Control group	3-year-olds	31	17/14	42.61 (3.23)	37–47
	4-year-olds	30	15/15	53.27 (3.16)	48–59
	5-year-olds	34	17/17	66.03 (3.42)	60–71
Empathic-concern group	3-year-olds	30	16/14	42.03 (1.75)	36–44
	4-year-olds	31	17/14	55.26 (3.01)	51–59
	5-year-olds	34	18/16	65.15 (3.08)	61–70
Moral-rule group	3-year-olds	30	16/14	42.67 (3.62)	37–47
	4-year-olds	34	17/17	54.18 (3.32)	48–59
	5-year-olds	33	17/16	66.76 (2.94)	63–71
Praise group	3-year-olds	33	15/18	42.12 (2.70)	36–47
	4-year-olds	30	15/15	54.80 (3.18)	48–59
	5-year-olds	32	16/16	65.44 (3.66)	60–71
Reward group	3-year-olds	33	16/17	41.79 (2.38)	36–47
	4-year-olds	32	14/18	54.00 (2.83)	48–58
	5-year-olds	30	14/16	63.67 (2.93)	60–69

### Helping Tasks and Procedure

Fabes et al.'s (1989) helping task was adapted and used to measure instrumental helping. This task involves acting on behalf of others who are unable to achieve their goals based on Warneken and Tomasello's (2009) definition.

The experimenter invited the preschoolers to listen to a story in a quiet room. When she was about to tell the story, she said that she had left her storybook outside the room and would leave to fetch the storybook. Meanwhile, she took out a photograph of a child of the same sex as the preschoolers. She said that the child was sick in a hospital, had nothing to play with, and liked playing a game with cards. She then took out two boxes and a large number (80 pieces) of red and green cards that were mixed together. She explained that the cards had to be put into corresponding boxes with red or green labels before the cards could be taken to the hospital for the sick child to play the game. To confirm that the preschoolers understood how to sort the cards, the experimenter presented one red and one green card and asked them how to sort the cards. All the preschoolers gave correct responses. The experimenter then emphasized different helping motivations for each group.

In the empathic-concern group, the experimenter said, “You can help the sick child in the hospital sort the cards. He (or she) is very miserable.” Similar expressions such as “the other child will be sad” were verified to emphasize emphatic concern (Malti et al., 2009b). In the moral-rule group, the experimenter said, “You can help the sick child in the hospital sort the cards. It is right to help others.” Similar expressions such as “it is not fair to steal,” were verified to emphasize moral rules (Malti et al., 2009b). Capraro et al. (2019) used similar moral frames, asking participants “what do you personally think is the morally right thing to do in this situation” before they made a choice in economic games. In the praise group, the experimenter said, “You can help the sick child in the hospital sort the cards. Teachers will praise kids who help.” “Teachers” were mentioned because in similar expressions related to praise and sanction, authority adults are always mentioned (Guttmann et al., 1985; Malti et al., 2009b). In the reward group, the experimenter said, “If you help the sick child in the hospital sort the cards, I will give you beautiful stickers.” Similar expressions were used to emphasize rewards (Guttmann et al., 1985). In the control group, the experimenter said only, “You can help the sick child in the hospital sort the cards” and did not emphasize any helping motivations.

Meanwhile, the experimenter emphasized to all the preschoolers that if they were not willing to help the sick children sort cards, they could play with toys in the room. The experimenter then left the room. The preschoolers could voluntarily choose what to do. Two minutes later, the experimenter returned and checked the cards and boxes to confirm whether the preschoolers helped. In the praise group, the preschoolers who helped were praised. In the reward group, the preschoolers who helped were rewarded, receiving beautiful stickers. In the other groups, the experimenter gave no responses to the preschoolers. The experimenter finally told a story to all the preschoolers. The number of cards that were correctly sorted by each preschooler was computed and represented their instrumental helping.

The preschoolers were tested individually in a quiet room in their preschools. After the experiment was completed, the experimenter gave five jigsaw puzzles to each class of preschoolers to thank those who participated in the study.

## Results

To indicate whether a specific helping motivation motivated instrumental helping, the helping motivation group had to be directly compared to the control group. To indicate whether the effect of helping motivation on instrumental helping (helping motivation vs. no helping motivation) changed with age, multiple age groups had to be included. Thus, a 2 (motivation: a helping motivation vs. no helping motivation) × 3 (age: 3 vs. 4 vs. 5 years) ANOVA was the most appropriate analysis and was conducted. The main effect of motivation indicated whether a specific helping motivation was effective. The interaction effect of motivation and age indicated whether the effect of a specific helping motivation changed with age.

**Figure 1:**
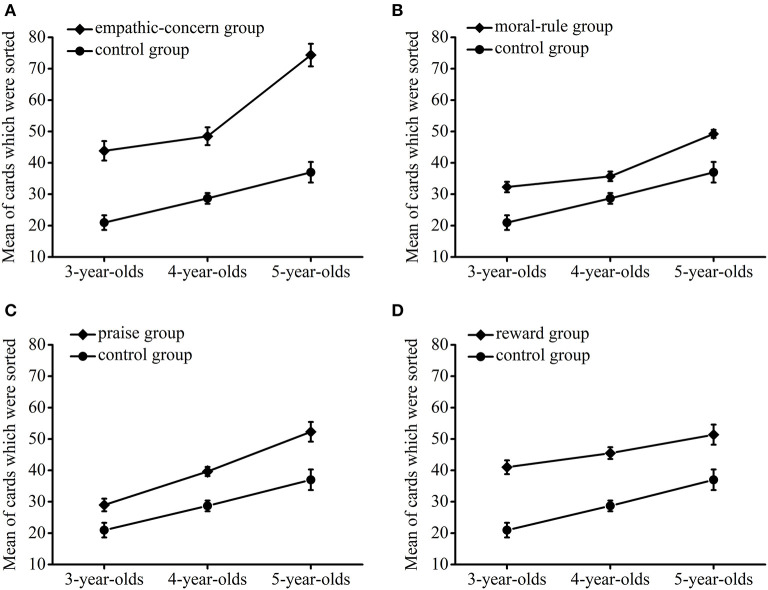
The number of cards sorted by a specific experimental group and the control group in the 3- to 5-year-olds. Error bars represent standard errors.

The number of cards sorted by the empathic-concern group and the control group in the 3–5-year-olds is shown in Figure 1A. A 2 × 3 ANOVA revealed a significant main effect of age, [*F*(2, 184) = 34.79], *p* < 0.001, η^2^ = 0.274. The main effect of motivation was significant, [*F*(1, 184) = 123.69], *p* < 0.001, η^2^ = 0.402. The empathic-concern group sorted more cards than the control group. More importantly, the interaction effect of motivation and age was significant, [*F*(2, 184) = 5.25], *p* = 0.006, η^2^ = 0.054. Simple effect analysis was then carried out. In the 3-year-olds, the empathic-concern group sorted more cards than the control group, *t* = 5.88, *p* < 0.001, *d* = 1.523. In the 4-year-olds, the result was similar, *t* = 5.98, *p* < 0.001, *d* = 1.574. In the 5-year-olds, although the result was also similar, the effect size was larger, *t* = 7.66, *p* < 0.001, *d* = 1.861, which explained why an interaction effect existed. Overall, empathic concern motivated the preschoolers to display instrumental helping, and the motivational effect of empathic concern increased greatly at the age of 5 years.

The number of cards sorted by the moral-rule group and the control group in 3–5-year-olds is shown in Figure 1B. A 2 × 3 ANOVA yielded a significant main effect of age, [*F*(2, 186) = 31.96], *p* < 0.001, η^2^ = 0.256. There was also a significant main effect of motivation, [*F*(1, 186) = 35.02], *p* < 0.001, η^2^ = 0.158. The moral-rule group sorted more cards than the control group. However, the interaction effect of motivation and age was not significant, [*F*(2, 186) = 0.89], *p* = 0.413, η^2^ = 0.009. These results indicated that moral rules motivated the preschoolers' instrumental helping, and the motivational effect of moral rules was equivalent among the 3–5-year-olds.

The number of cards sorted by the praise group and the control group in the 3–5-year-olds is shown in Figure 1C. A 2 × 3 ANOVA found a significant main effect of age, [*F*(2, 184) = 32.56], *p* < 0.001, η^2^ = 0.261. The main effect of motivation was also significant, [*F*(1, 184) = 31.97], *p* < 0.001, η^2^ = 0.148. The praise group sorted more cards than the control group. Likewise, the interaction effect of motivation and age was not significant, [*F*(2, 184) = 1.12], *p* = 0.330, η^2^ = 0.012. These results suggested that praise motivated the preschoolers to display instrumental helping, and the motivational effect of praise was similar among the 3–5-year-olds.

The number of cards sorted by the reward group and the control group in the 3–5-year-olds is shown in Figure 1D. In a 2 × 3 ANOVA, age had a significant main effect, [*F*(2, 184) = 13.82], *p* < 0.001, η^2^ = 0.131. The main effect of motivation was significant, [*F*(1, 184) = 68.49], *p* < 0.001, η^2^ = 0.271. The reward group sorted more cards than the control group. Nevertheless, the interaction effect of motivation and age was not significant, [*F*(2, 184) = 0.65], *p* = 0.526, η^2^ = 0.007. Therefore, rewards motivated the preschoolers' instrumental helping, and the motivational effect of rewards was steady from the age of 3–5 years.

## Discussion

Previous studies have often investigated prosocial motivations through children's answers about why an action is right or why an action leads to certain emotions (Malti et al., 2009a,b; Sengsavang et al., 2015). They lack systematic theoretical frameworks to organize various prosocial motivations and rely more on children's subjective reports. The present study examined the motivations for preschoolers' instrumental helping under Eisenberg et al.'s (2016) model of prosocial motivations. Specific helping motivation was emphasized for each experimental group but not the control group. The preschoolers' instrumental helping was then measured. The causal effects of various helping motivations on instrumental helping were thus revealed in the present study.

Based on Eisenberg et al.'s (2016) model, the present study investigated what motivates preschoolers to display instrumental helping and how various helping motivations develop with age. As hypothesized, the empathic-concern group helped the sick child sort more cards than the control group. Empathic concern motivated the preschoolers to display instrumental helping. Previous studies have also demonstrated that when another person is upset or harmed, young children show empathic concern and subsequent prosocial behavior (Vaish et al., 2009; Williams et al., 2014). Du and Hao (2018) found that moral stories emphasizing an actor's negative emotion toward his or her non-helping behavior facilitated preschoolers' donating behavior. This result might also be explained by preschoolers' empathy for the actor. A recent study revealed preschoolers' empathy-related electrophysiological responses to pictures of people in pain (Decety et al., 2018). In addition, Hoffman (1982) proposed a series of developmental stages of empathy from infancy. Although infants have been observed displaying empathic responses (Liddle et al., 2015; Abramson et al., 2018), Hoffman claimed that by 2–3 years, “with the beginning of role taking, empathy becomes an increasingly veridical response to the other's feelings in the situation” (Hoffman, 1982, p. 94). This is consistent with our finding that empathic concern motivated the 3–5-year-olds' instrumental helping. Our results also indicated that the motivational effect of empathic concern was much stronger in 5-year-olds. A recent study found that empathy develops significantly at the age of 5 years (Brown et al., 2017). This may explain why this altruistic motivation increased greatly at the age of 5 years in the present study.

Moral rules also motivated the preschoolers to display instrumental helping, which is consistent with our hypotheses. The moral rule that “it is right to help others” reflected a care orientation. Cassidy et al. (1997) found that 3–5-year-olds can use a care orientation to solve moral dilemmas. However, our results did not support the hypothesis that this altruistic motivation increases with age. The motivational effect of moral rules was equivalent among the 3–5-year-olds in the present study. Moreover, the motivational effect of moral rules was relatively weaker. Using moral rules to guide behavior might be a difficult process for both younger and older preschoolers because in the process preschoolers need to display specific behaviors based on general rules. Eisenberg et al. (2016) mentioned that adolescence is more related to moral rules and principles. During adolescence, moral identity rapidly develops (Hardy and Carlo, 2005; Moshman, 2011). “Moral identity is presumed to reflect widely endorsed moral ideals and principles” (Aquino and Reed, 2002, p. 1431) and is closely associated with prosocial behavior (Hardy et al., 2015; Patrick et al., 2018). Thus, the motivational effect of moral rules may significantly increase in adolescence. With regard to adults, previous studies indicate that morally loaded language affects adults' prosocial behavior (Capraro and Vanzo, 2019). Adults who take actions framed as moral are more prosocial in subsequent choices (Capraro and Rand, 2018; Tappin and Capraro, 2018). In addition, asking adults what they think is the morally right thing to do increases their prosocial behavior over time and across contexts (Capraro et al., 2019). These studies indicate that morality preference strongly motivates adults to display prosocial behavior. Generalized or abstract moral principles are formed at a mature age (Kohlberg, 1969). Thus, compared to preschoolers, the morality of actions may be more salient to adults and valued by them.

With regard to praise, the hypotheses were partially supported. As expected, the effect of praise on instrumental helping was positive. This result is consistent with those of Guttmann et al. (1985). However, the motivational effect of praise was similar for the 3–5-year-olds, which is inconsistent with the hypotheses. First, in the present study, the expected praise was given by teachers as authority adults. Thus, both younger and older preschoolers might have valued and been motivated by this type of praise. Second, Henderlong and Lepper (2002) disagreed with a behavioral definition of praise as verbal reinforcement because praise can affect children's inner states. Adults also praise children in everyday life and expect it to increase their self-esteem (Henderlong and Lepper, 2002). Because positive environments are beneficial for self-esteem (Orth, 2018; Krauss et al., 2020), praise as an approval from the environment may make children feel good about themselves. Self-esteem levels increase from the age of four (Orth et al., 2018), which means that self-esteem is likely to become important before the age of 5 years. Therefore, praise motivates both younger and older preschoolers to provide instrumental help, bringing them positive feelings about themselves.

The motivational effect of rewards on instrumental helping supported our hypotheses but is inconsistent with the findings of some previous studies (Warneken and Tomasello, 2008; Ulber et al., 2016). In the present study, when rewards were emphasized to the preschoolers, they displayed more instrumental helping. Behaviorists believe that rewards are important determinants of helping (Bar-Tal, 1976). In contrast, in Warneken and Tomasello (2008) and Ulber et al. (2016), children were first induced to attribute their prosocial behavior to a desire for rewards, and then their prosocial behavior was undermined. These researchers used the “overjustification” hypothesis to explain the negative effect of rewards. Because the preschoolers were not induced to make such an attribution in the present study, rewards show a positive effect. With regard to the developmental trends, the motivational effect of rewards did not decrease with age but developed steadily. In other words, rewards motivated preschoolers of different ages to display instrumental helping to a similar extent. Previous studies indicate that hedonistic reasoning decreases from the end of the preschool years to elementary school years (Eisenberg et al., 1983, 1987). Taken together, these results imply that with increasing age, although preschoolers mention personal gains less often, their behavior is still affected by consideration of personal gains. Flavell (1968) indicated that a genuine shift from an egocentric orientation occurs at an older age, the age of 7 years.

In addition to the effect of motivations on instrumental helping, the present study found an age effect on instrumental helping. This typical prosocial behavior increased from the ages of 3 to 5. Previous studies have also found similar age trends in the preschool years (Hart et al., 1992; Persson, 2005; Gummerum et al., 2010; Van Berkel et al., 2015) and further confirmed that age trends continued to exist after the preschool years (Cowell et al., 2017). As the present study showed, empathic concern as an important altruistic motivation significantly develops during the preschool years. This result might explain the increase in instrumental helping with age. Consistent with this explanation, previous studies also indicate that empathic concern and emotion attribution primarily affect the development of preschoolers' prosocial behavior (Gummerum et al., 2010; Williams et al., 2014).

In summary, although previous studies have confirmed the existence of multiple motivations for prosocial behavior, young children's prosocial motivations have not been systematically investigated. Their prosocial motivations are usually separately investigated and defined as intrinsic or extrinsic motivations (Warneken and Tomasello, 2008; Hepach et al., 2013, 2017; Ulber et al., 2016). However, intrinsic or extrinsic motivations may not reflect egocentric or other-centric orientations. Eisenberg et al.'s (2016) model provides a theoretical framework for investigating young children's prosocial motivations in terms of egoistic vs. altruistic motivations, reflecting the essence of motivations to a larger extent. Based on that model, the present study contributes to our understanding of preschoolers' prosocial motivations in terms of the essence of motivations. In addition, the present study contributes to our knowledge of the development of various motivations during the preschool years: generally, altruistic motivations develop more quickly than egoistic motivations, and affective altruistic motivations develop more quickly than cognitive altruistic motivations.

Some limitations of the present study should be noted. First, the present study focused on the effects of various helping motivations and their developmental trends during the preschool years. Nevertheless, the preschool years involve a narrow age range. How various helping motivations develop from early childhood to middle childhood is not clear and may be more valuable to investigate. Future studies need to extend the research population to school-age children. Second, based on Eisenberg et al.'s (2016) model, the present study examined only prosocial motivations that are typical for preschoolers. Motivations such as social relatedness and reducing aversive arousal were not investigated and should be considered in future studies. Third, the present study investigated the effects of various motivations only on helping behavior. The effects of various motivations on other important prosocial behaviors, such as sharing, donating, and comforting, are unclear. It is reasonable to assume that different types of prosocial behavior are conducted due to different motivation. Future studies should investigate this issue.

In conclusion, preschoolers help others due to a variety of altruistic and egoistic motivations, including empathic concern, adherence to moral rules, obtaining praise and rewards. Most of the helping motivations develop steadily during the preschool years, but empathic concern as an altruistic motivation increases greatly at the end of the preschool years. The present study thus reveals the diversity of preschoolers' helping motivations with Eisenberg et al.'s model of prosocial motivations.

## Data Availability Statement

The raw data supporting the conclusions of this article will be made available by the authors, without undue reservation.

## Ethics Statement

The studies involving human participants were reviewed and approved by the Research Ethics Board of the School of Psychology of Capital Normal University. Written informed consent to participate in this study was provided by the participants' legal guardian/next of kin.

## Author Contributions

JH contributed to conception of the study and wrote the draft of the manuscript. XD designed the study and performed the statistical analysis. Both authors contributed to the article and approved the submitted version.

## Conflict of Interest

The authors declare that the research was conducted in the absence of any commercial or financial relationships that could be construed as a potential conflict of interest.
